# Methane production from protozoan endosymbionts following stimulation of microbial metabolism within subsurface sediments

**DOI:** 10.3389/fmicb.2014.00366

**Published:** 2014-08-06

**Authors:** Dawn E. Holmes, Ludovic Giloteaux, Roberto Orellana, Kenneth H. Williams, Mark J. Robbins, Derek R. Lovley

**Affiliations:** ^1^Department of Microbiology, University of MassachusettsAmherst, MA, USA; ^2^Physical and Biological Sciences, Western New England UniversitySpringfield, MA, USA; ^3^Lawrence Berkeley National LaboratoryBerkeley, CA, USA

**Keywords:** anaerobic protozoa, methanogenesis, *in situ* transcriptomics, uranium bioremediation, endosymbiont

## Abstract

Previous studies have suggested that protozoa prey on Fe(III)- and sulfate-reducing bacteria that are enriched when acetate is added to uranium contaminated subsurface sediments to stimulate U(VI) reduction. In order to determine whether protozoa continue to impact subsurface biogeochemistry after these acetate amendments have stopped, 18S rRNA and ß-tubulin sequences from this phase of an *in situ* uranium bioremediation field experiment were analyzed. Sequences most similar to *Metopus* species predominated, with the majority of sequences most closely related to *M. palaeformis*, a cilitated protozoan known to harbor methanogenic symbionts. Quantification of *mcrA* mRNA transcripts in the groundwater suggested that methanogens closely related to *Metopus* endosymbionts were metabolically active at this time. There was a strong correlation between the number of *mcrA* transcripts from the putative endosymbiotic methanogen and *Metopus* ß-tubulin mRNA transcripts during the course of the field experiment, suggesting that the activity of the methanogens was dependent upon the activity of the *Metopus* species. Addition of the eukaryotic inhibitors cyclohexamide and colchicine to laboratory incubations of acetate-amended subsurface sediments significantly inhibited methane production and there was a direct correlation between methane concentration and *Metopus* ß-tubulin and putative symbiont *mcrA* gene copies. These results suggest that, following the stimulation of subsurface microbial growth with acetate, protozoa harboring methanogenic endosymbionts become important members of the microbial community, feeding on moribund biomass and producing methane.

## Introduction

Methanogenic microbial communities exemplify the importance of interspecies interactions. These include not only the various forms of interspecies electron transfer between bacteria and methanogens (Stams and Plugge, 2009; Malvankar and Lovley, 2014; Rotaru et al., 2014), but also the symbiotic association of protozoa and endosymbiotic methanogens (van Hoek et al., 2000; Fenchel and Finlay, 2010). Endosymbiotic methanogens make significant contributions to methane production in many environments including marine sediments (Fenchel, 1993), anaerobic landfills (Finlay and Fenchel, 1991), recently flooded rice paddy soils (Schwarz and Frenzel, 2005), wastewater reactors (Narayanan et al., 2007; Priya et al., 2008) and the rumen (Newbold et al., 1995; Ushida et al., 1997).

The majority of methanogenic endosymbionts are associated with ciliated protozoa, but methanogens have also been found in the cytoplasm of anaerobic amoebae and flagellates (Vogels et al., 1980; Vanbruggen et al., 1983; van Hoek et al., 2006; Nowack and Melkonian, 2010; Hackstein, 2011). Methanogen-harboring ciliates contain specialized organelles called hydrogenosomes that ferment pyruvate, forming acetate, H_2_ and CO_2_ (Yarlett and Hackstein, 2005; Fenchel and Finlay, 2010). The acetate formed by this fermentation reaction is then used by the ciliate as an energy and carbon source, while the symbiont can utilize the H_2_ and CO_2_ for methanogenesis.

Recent studies have emphasized the importance of protozoa in influencing microbial growth and activity in uranium-contaminated aquifers in which microbial U(VI) reduction is stimulated with the addition of organic electron donors (Holmes et al., 2013). The addition of acetate to groundwater promotes the activity of bacteria such as *Geobacter* species that reduce highly soluble U(VI) to less soluble U(IV) (Anderson et al., 2003; Wall and Krumholz, 2006; Wu et al., 2007; Williams et al., 2011) and the growth of *Geobacter* populations is followed by a bloom of protozoa that feed on the *Geobacter* (Holmes et al., 2013). With continued addition of acetate, sulfate-reducing bacteria also increase in abundance (Vrionis et al., 2005; Miletto et al., 2011), specifically inducing the growth of a different family of protozoa that appear to specialize in predation of the sulfate reducers (Holmes et al., 2013).

In addition to the benefit of precipitating uranium from contaminated groundwater, stimulating anaerobic respiration in the subsurface may have unintended negative consequences. For example, as U(VI) was reductively precipitated from the groundwater, arsenic was also released (Giloteaux et al., 2013), presumably as the result of microbial reduction of Fe(III) minerals that adsorb arsenic in the subsurface (Dowdle et al., 1996; Redman et al., 2002; Islam et al., 2004; Rowland et al., 2007; Hery et al., 2010; Giloteaux et al., 2013). It has already been shown that high levels of organic contaminants in groundwater can promote methanogenesis (Lovley, 1997; Bekins et al., 2001; Kleikemper et al., 2005). Therefore, it might be expected that long-term acetate additions to the subsurface could also promote methanogenesis by providing a substrate for growth of acetoclastic methanogens, or indirectly from the subsequent degradation of the biomass of acetate-oxidizing microorganisms that accumulates in the subsurface (N'Guessan et al., 2008; Wrighton et al., 2012, 2014; Hug et al., 2013). Methane production during *in situ* uranium bioremediation is undesirable because methane may reduce hydraulic transmissivity, disrupting the delivery of electron donor to contaminated zones. Furthermore, the production of methane, a strong greenhouse gas, has a negative impact on the overall environmental benefit of the bioremediation process.

Therefore, in our continuing investigation of the impact of protozoa on *in situ* bioremediation of uranium-contaminated groundwater, the potential for methanogen-harboring ciliates to contribute to methane production was investigated. The results suggest that this could be a source of methane production in the subsurface after acetate amendments have been discontinued.

## Materials and methods

### Site and description of field site

In 2011, a small-scale *in situ* bioremediation experiment was conducted on the grounds of a former uranium ore processing facility in Rifle, Colorado (USA) during the months of August-October as previously described (Giloteaux et al., 2013). Subsurface microbial activity was stimulated by acetate additions during the months of August-October in a manner consistent with previous such experiments at the site (Anderson et al., 2003; Vrionis et al., 2005; Williams et al., 2011). The monitoring array consisted of an injection gallery with 6 injection wells, 9 down-gradient wells, and 1 background monitoring well located upstream from the injection gallery (See Supplementary Material, Figure S1). Groundwater for the experiments was collected from well CD-01.

During the field experiment, a concentrated acetate/bromide solution (150/20 mM) mixed with native groundwater was injected into the subsurface to provide approximately 15 mM acetate to the groundwater over the course of 68 days as previously described (Anderson et al., 2003; Williams et al., 2011). Bromide was utilized as a non-reactive tracer to enable injectate delivery to down-gradient monitoring locations.

### Rifle sediment incubations and enrichment cultures

Background subsurface sediments were collected near the acetate-injection test plot with a backhoe, placed in sealed mason jars, and stored at 16°C until use. Unfiltered background groundwater for sediment incubations was pumped to the surface into 5-gallon carboys with a peristaltic pump and stored at 4°C.

For sediment incubations, 40 g of the background sediments described above, 16 ml groundwater and acetate (~20 mM) were added to 60 ml serum bottles in an anaerobic chamber under an N_2_ atmosphere and incubated at 18°C. Six acetate-amended (3 with eukaryotic inhibitors cycloheximide and colchicine; final concentration 200 mg/L each) and 3 control (no acetate additions) incubations were monitored over the course of 50 days. Fe(III) reduction, sulfate reduction, or methanogenesis was not observed in control incubations.

### Analytical techniques

Groundwater samples for geochemical analyses were collected after purging 12 l of groundwater from the wells with a peristaltic pump. Ferrous iron was measured spectrophotometrically immediately after sampling using the phenanthroline method (AccuVac ampules; Hach Company) for ferrous iron. After filtration through a 0.2 μm pore size polytetrafluoroethylene [PTFE (Teflon)] filter (Alltech Associates, Inc., Deerfield, IL), acetate, bromide, chloride, sulfate, and thiosulfate were measured using an ion chromatograph (ICS-2100, Dionex, CA) equipped with an AS18 column under isocratic elution with 32 mM KOH as the eluent.

Samples for dissolved gas analysis were collected using a passive gas sampler consisting of a 5 cm length of gas permeable silicone tubing affixed to the end of a 10 mL valve sealable, gas-tight syringe (Valco Instruments Co. Inc.; Baton Rouge, LA) patterned on that described by Spalding and Watson (Spalding and Watson, 2006). Syringes were affixed to rigid tubing and emplaced within the monitoring wells (CU01, CD01) at a depth of ca. 5 m below top of casing. Groundwater elevations at the time of sampling were recorded at each well in order to calculate the hydrostatic pressure associated with each measurement time point. Partitioning of dissolved gases in groundwater across the permeable silicon tubing results in equilibration of gases within the syringe over a period of 2–3 days (Spalding and Watson, 2006) thus reflecting an averaged value between sampling time points. Prior to analysis, syringes were removed from the well bores and the gas-tight valves closed before being analyzed on site via gas chromatography (GC) using an SRI Model 8610 GC equipped with multiple detectors: a helium ionization detector (HID) running in parallel with a thermal conductivity detector (TCD) discharging to a reductive gas detector (RGD). Helium (HID) and argon (TCD, RGD) were used as the carrier gases for the detectors, as indicated, with gas volumes of 1 mL and 9 mL used for the HID and TCD/RGD, respectively. Gas concentrations in the aqueous phase were calculated as described (Spalding and Watson, 2006) using a methane gas solubility of 1.23 × 10^−3^ mol/L/atm.

In the laboratory sediment incubations Fe(II) was monitored over time with a ferrozine assay in a split-beam dual-detector spectrophotometer (Spectronic Genosys2; Thermo Electron Corp., Mountain View, CA) at an absorbance of 562 nm after a 1 h extraction with 0.5 N HCl (Lovley et al., 1987; Lovley and Phillips, 1988). The remaining Fe(III) in the sediments that was not HCl-extractable was then converted to Fe(II) with the addition of 0.25 M hydroxylamine (Lovley et al., 1987). After addition of hydroxylamine, samples were incubated for an additional hour, and then measured with the ferrozine assay. Methane in the headspace of sediment incubations was measured by gas chromatography with a flame ionization detector (Shimadzu, GC-8A), and hydrogen sulfide concentrations were determined by the methylene blue method (Truper and Schlegel, 1964).

### Extraction of nucleic acids from samples

DNA and RNA were extracted from groundwater collected from the U(VI) contaminated aquifer during the bioremediation field experiments. In order to obtain sufficient biomass from the groundwater, it was necessary to concentrate 50 l of groundwater by impact filtration on 293 mm diameter Supor membrane disc filters with pore sizes of 1.2 and 0.2 μm (Pall Life Sciences), which took about 3 min. All filters were placed into whirl-pack bags, flash frozen in a dry ice/ethanol bath, and shipped back to the laboratory where they were stored at −80°C. RNA was extracted from filters as previously described (Holmes et al., 2005) and DNA was extracted with the FastDNA SPIN Kit for Soil (MP Biomedicals, Santa Ana, CA). DNA was also extracted from groundwater collected from the sediment incubations with the FastDNA SPIN Kit for Soil, however, it was not necessary to concentrate samples on membrane disc filters.

Analysis of nucleic acids by spectrophotometry (NanoDrop, Thermo Scientific), microfluidic analysis (Experion, BioRad), and gel electrophoresis showed that high quality DNA and RNA were extracted from the groundwater samples. In order to ensure that RNA samples were not contaminated with DNA, PCR amplification with primers targeting the 16S rRNA gene was conducted on RNA samples that had not undergone reverse transcription.

A DuraScript enhanced avian RT single strand synthesis kit (Sigma) was used to generate cDNA as previously described (Holmes et al., 2005).

### PCR amplification parameters and clone library construction

Several previously described primer pairs were used for amplification of 16S bacterial and archaeal rRNA, 18S rRNA, *mcrA*, and ß-tubulin gene fragments from genomic DNA and cDNA constructed from mRNA extracted from groundwater. Gene fragments from the bacterial 16S rRNA gene were amplified with 8F (Eden et al., 1991) and 519R (Lane et al., 1985); 344F and 915R (Casamayor et al., 2002) amplified archaeal 16S rRNA gene fragments; 515F (Giovannoni et al., 1988) and 1209R (Reysenbach et al., 1992) amplified eukaryotic 18S rRNA gene fragments; BT107F and BT261R (Baker et al., 2004) amplified protozoan ß-tubulin gene fragments; and MLf (Luton et al., 2002) and ME2 (Juottonen et al., 2006) amplified *mcrA* gene fragments (See Supplementary Material, Table S1). The 18S rRNA and ß-tubulin primer sets were both non-specific and amplified both protozoan and non-protozoan eukaryotic gene sequences. Some of the non-protozoan gene sequences detected at this site came from plant, fungal, and animal species which accounted for ca. 5 and 15% of the 18S rRNA and ß-tubulin clone libraries. This study focused exclusively on the protozoan sequences detected in these eukaryotic libraries.

A 50 μl PCR reaction consisted of the following solutions: 10 μl Q buffer (Qiagen), 0.4 mM of each dNTP, 1.5 mM MgCl_2_, 0.2 μM of each primer, 5 μg bovine serum albumin (BSA), 2.5 U Taq DNA polymerase (QIAGEN) and 10 ng of DNA template. Amplification was performed with a minicycler PTC 200 (MJ Research) starting with 5 min at 94°C, followed by 35 cycles consisting of denaturation (45 s at 94°C), annealing (see Table S1), extension (90 s at 72°C), and a final extension at 72°C for 10 min.

After PCR amplification of these gene fragments, PCR products were purified with the Gel Extraction Kit (Qiagen), and cloned into the TOPO TA cloning vector, version M (Invitrogen, Carlsbad, CA). One hundred plasmid inserts from each of these clone libraries were sequenced with the M13F primer at the University of Massachusetts Sequencing Facility.

### Testing and design of qPCR primers

Quantitative PCR primer sets targeting the ß-tubulin gene from *in situ Metopus* species and the *mcrA* gene from *in situ* Methanomicrobiales species were designed according to the manufacturer's specifications (Applied Biosystems) and had amplicon sizes ranging from 100 to 200 bp (See Supplementary Table S1). The *Metopus* specific primer set (Met-bt_60f/155r) was designed from *Metopus* ß-tubulin clone 9 (KJ609554) which accounted for 90% of the sequences from the ß-tubulin cDNA clone library assembled with groundwater collected on day 95 of the field experiment and shared 99% of its nucleotides with the ß-tubulin gene from *Metopus palaeformis*. The Methanomicrobiales specific primer set (Rifle_mcrA_379f/489r) was designed from Rifle *mcrA* clone 6 (KJ609576) and accounted for 52% of the *mcrA* cDNA clone library assembled with groundwater collected on day 95.

### Quantification of gene and transcript abundance by qPCR

Quantitative PCR amplification and detection were performed with the 7500 Real Time PCR System (Applied Biosystems) using genomic DNA and cDNA made by reverse transcription from mRNA extracted from groundwater collected during the bioremediation experiment. All qPCR assays had triplicate biological and technical replicates. Each reaction mixture consisted of a total volume of 25 μL and contained 1.5 μL of the appropriate primers (stock concentrations, 1.5 μM), 5 ng cDNA, and 12.5 μL Power SYBR Green PCR Master Mix (Applied Biosystems). Standard curves covering 8 orders of magnitude were constructed with serial dilutions of known amounts of purified cDNA quantified with a NanoDrop ND-1000 spectrophotometer at an absorbance of 260 nm. Transcript abundances and qPCR efficiencies (90–99%) were calculated from appropriate standard curves and all qPCR experiments followed MIQE guidelines (Bustin et al., 2009). Optimal thermal cycling parameters consisted of an activation step at 50°C for 2 min, an initial 10 min denaturation step at 95°C followed by 40 cycles of 95°C for 15 s and 58–60°C for 1 min. After 40 cycles of PCR amplification, dissociation curves were made for all qPCR products by increasing the temperature from 58 to 95°C at a ramp rate of 2%. The curves all yielded a single predominant peak, further supporting the specificity of the PCR primer pairs.

### Phylogenetic analysis

16S and 18S rRNA and functional gene sequences were assembled with Geneious 5.6 and compared to GenBank nucleotide and protein databases with the blastn and blastx algorithms (Altschul et al., 1998). Alignments were made in ClustalX (Thompson et al., 1997) and corrected with ProSeq v2.9 (Filatov, 2002) before phylogenetic trees were constructed with Mega v6 (Tamura et al., 2013). The Maximum likelihood algorithm with the Nearest-Neighbor Interchange was used to construct all phylogenetic trees. All evolutionary distances were computed with the Tamura-Nei substitution model (Tamura and Nei, 1993) with 100 bootstrap replicates.

The nucleotide sequences of 18S rRNA, ß-tubulin, and 16S rRNA genes amplified from the uranium-contaminated aquifer have been deposited in the GenBank database under accession numbers KJ609533-KJ609576.

## Results

### Methane production following injection of acetate into the subsurface

**Figure 1:**
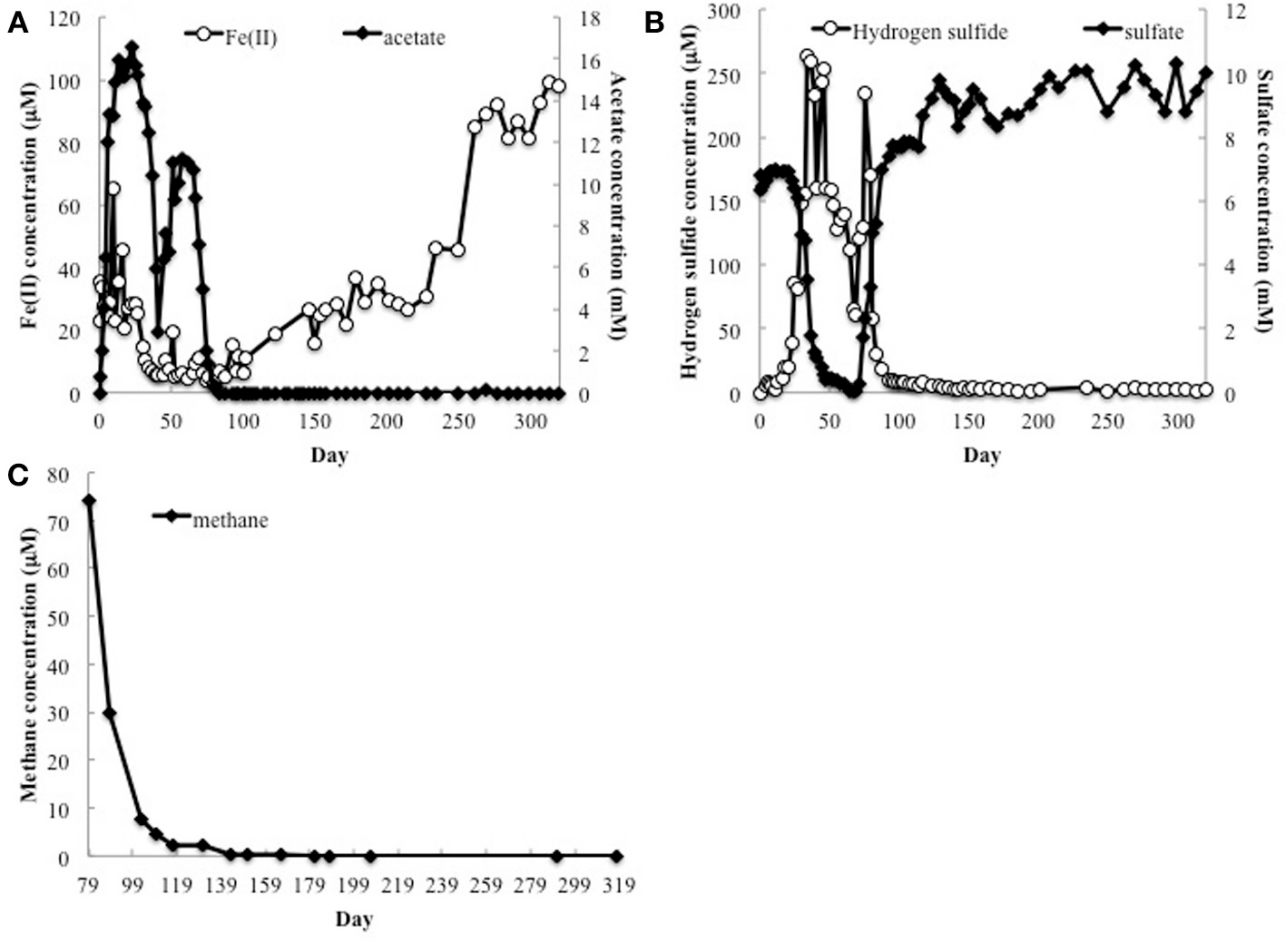
**(A)** Fe(II), acetate, **(B)** sulfate and H_2_S concentrations in groundwater collected from well CD-01 over the course of 320 days. **(C)** Methane concentrations in the subsurface starting 79 days after initial acetate injections (day 0).

Acetate was pumped into the subsurface for 68 days to promote *in situ* uranium bioremediation. The initial accumulation of Fe(II), followed by an accumulation of sulfide, indicated a typical succession (Anderson et al., 2003; Vrionis et al., 2005) of Fe(III) reduction followed by sulfate reduction in response to the acetate amendment (Figures 1A,B). Although acetate was no longer being pumped into the subsurface after day 68, low concentrations of acetate continued to be detected until day 88 (Figure 1A).

Methane analysis initiated on day 79 detected methane in the groundwater, which declined over time, but remained detectable well after acetate had been depleted (Figure 1C). Methane was not detected (detection limit 0.1 μM) in the groundwater up-gradient of the acetate injection site.

### Protozoa known to harbor methanogenic endosymbionts detected in groundwater

**Figure 2:**
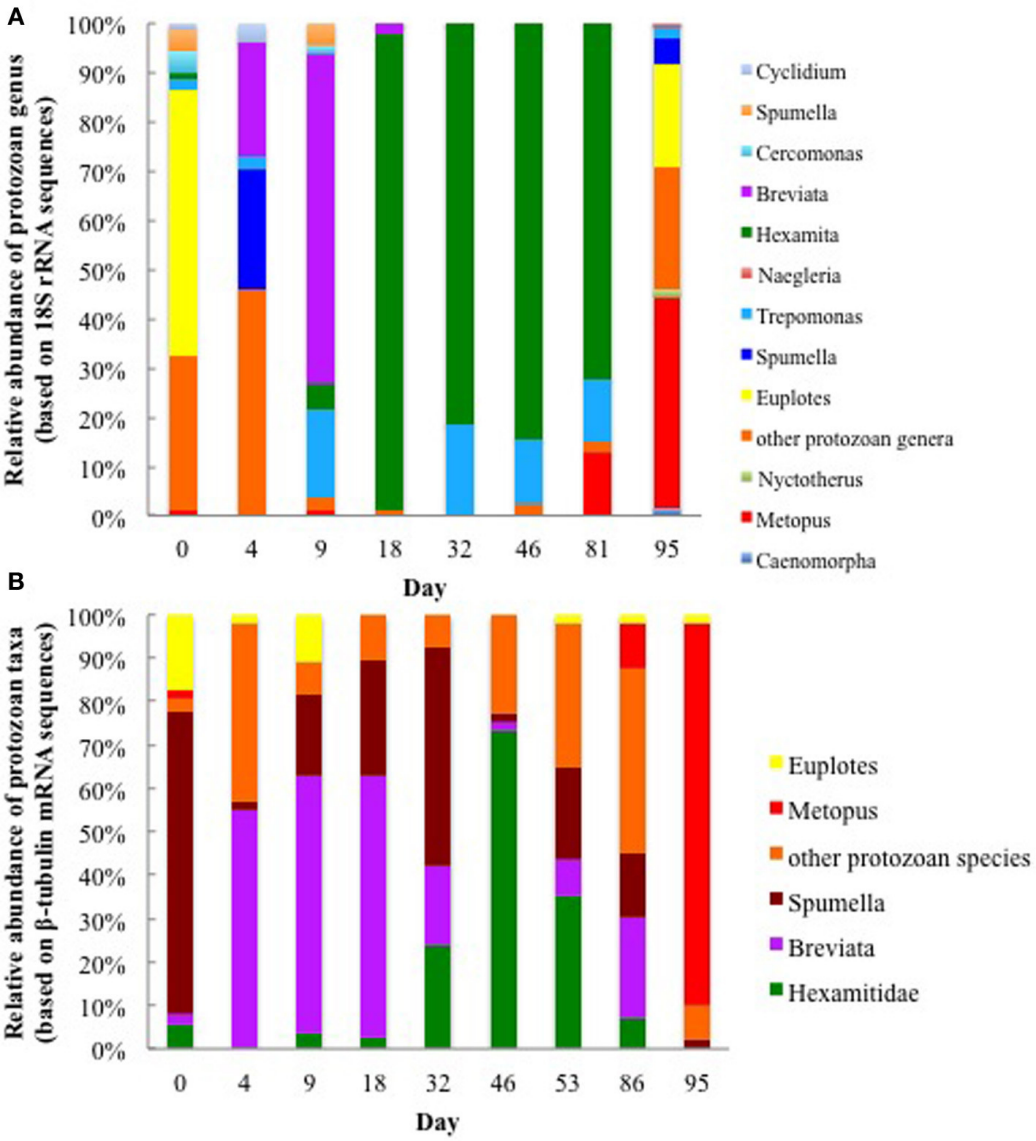
**Relative abundance of various protozoan taxa found in groundwater collected on various days during the 2011 field experiment based on (A) protozoan 18S rRNA transcripts and (B) ß-tubulin mRNA transcripts**.

As previously reported (Holmes et al., 2013), the Fe(III) reduction phase of the bioremediation process was associated with the growth of *Geobacter* species and a specific enrichment of protozoa from the genus *Breviata*. The subsequent growth of sulfate-reducing bacteria was accompanied by a bloom in protozoa from the family Hexamitidae (Holmes et al., 2013). Following the Fe(III) and sulfate reducing phases of the experiment, when acetate concentrations were negligible, 18S rRNA and ß-tubulin mRNA transcripts most similar to *Metopus* species (Figure 2) as well as *Metopus* ß-tubulin gene copies (Supplementary Figure S2), increased dramatically. These results demonstrated that *Metopus*, which are ciliated protozoa known to harbor methanogens, became predominant members of the protozoan community.

The majority (42 and 88%) of the *Metopus* 18S rRNA and ß-tubulin mRNA transcript sequences were most similar to *M. palaeformis* (96 and 99% identical respectively; represented by *Metopus* clones C and 9) (Supplementary Figures S3, S4). Sequences from other methanogen harboring ciliates from the genera *Cyclidium*, *Nyctotherus*, and *Caenomorpha* were also detected but never accounted for more than 4% of the protozoan community, making it unlikely that they made a significant contribution to methane production in the subsurface.

### Methanogen endosymbiont sequences detected in the groundwater

**Figure 3:**
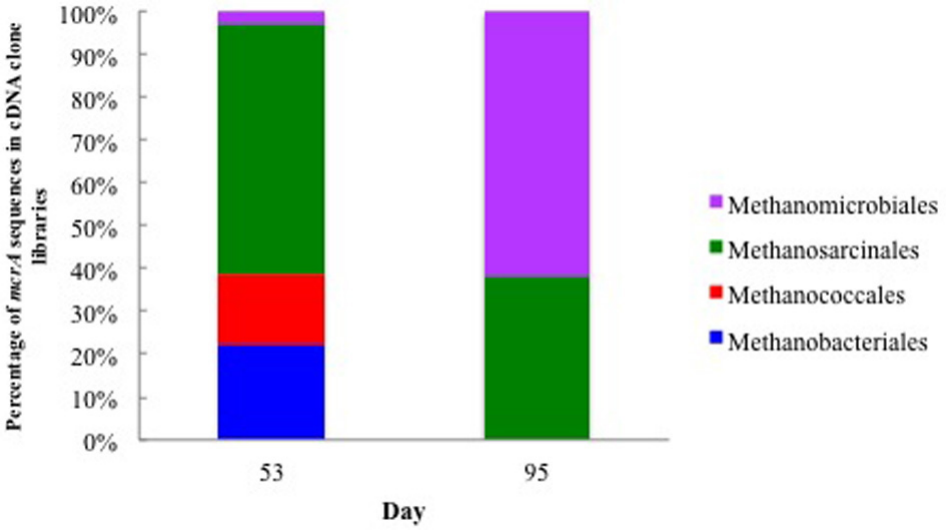
**Relative abundance of *mcrA* mRNA transcripts from methanogens associated with groundwater collected on day 53 when acetate concentrations were 6.71 mM and day 95 when acetate concentrations were below detection**.

In addition to expression of genes specific to methanogen-harboring protozoa in the groundwater during the 2011 field experiment, a gene specific to methanogens, *mcrA* (methyl coenzyme M reductase A), was also being actively transcribed in the subsurface following the sulfate reducing phase of the experiment. When acetate concentrations were high, the majority of *mcrA* mRNA transcripts (58.3%) clustered with Methanosarcinales (Figure 3). However, when acetate concentrations dropped below detectable levels on day 95, 60% of the *mcrA* transcripts clustered with Methanomicrobiales, an order that is frequently associated with methanogen-harboring ciliates found in freshwater environments (Fenchel and Finlay, 2010). The dominant Methanomicrobiales sequence (*mcrA* clone 6; 52% of the cDNA clone library) shared 87% of its nucleotides with an endosymbiont of *Metopus contortus* (Supplementary Figure S5) (Vanbruggen et al., 1983).

### Correlation between *Metopus* and Methanomicrobiales activity in uranium contaminated subsurface sediments

**Figure 4:**
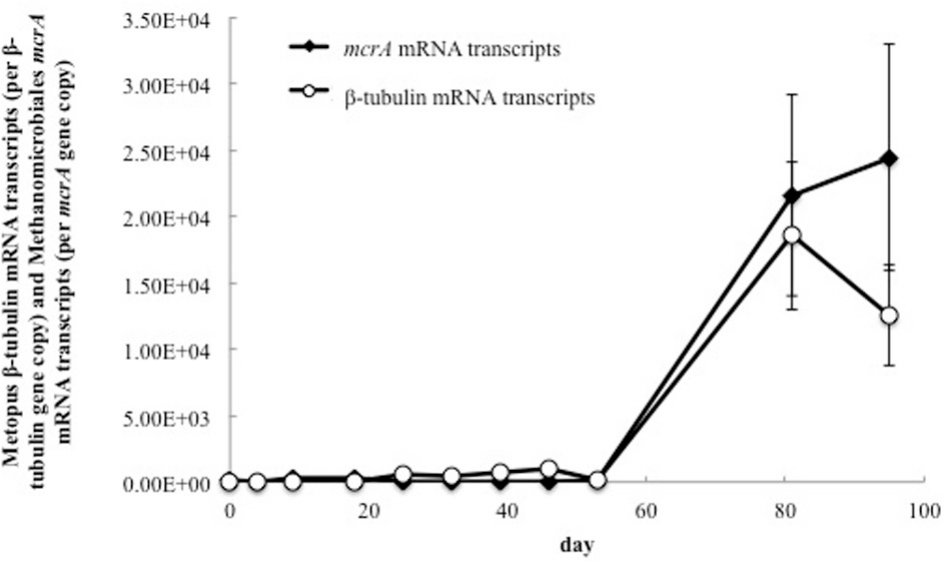
**Quantitative RT-PCR analysis of mRNA transcripts from Methanomicrobiales *mcrA* and *Metopus* ß-tubulin genes in groundwater collected from well CD-01 over the course of the field experiment**. The number of mRNA transcripts per μg total RNA were normalized against the number of gene copies per μg total DNA.

In order to evaluate the association of the dominant putative methanogenic symbiont (represented by *mcrA* clone 6) and the dominant *Metopus* species (represented by ß-tubulin clone 9), transcripts from methanogen- and protozoan-related genes were quantified over time (Figure 4). There was a strong correlation (Pearson's correlation, *r* = 0.95, *p* = 0.005) between the number of transcripts from clone 6 *mcrA* and *Metopus* ß-tubulin genes during the course of the field experiment. Transcription of both genes was low (2.04 × 10^1^ − 9.6 × 10^2^ mRNA transcripts per gene copies) until day 81, when the number of mRNA transcripts increased by two orders of magnitude (Figure 4). These results suggest that the growth and activity of this Methanomicrobiales species was directly related to the growth and activity of this *Metopus* protozoan species.

### Laboratory sediment incubations

**Figure 5:**
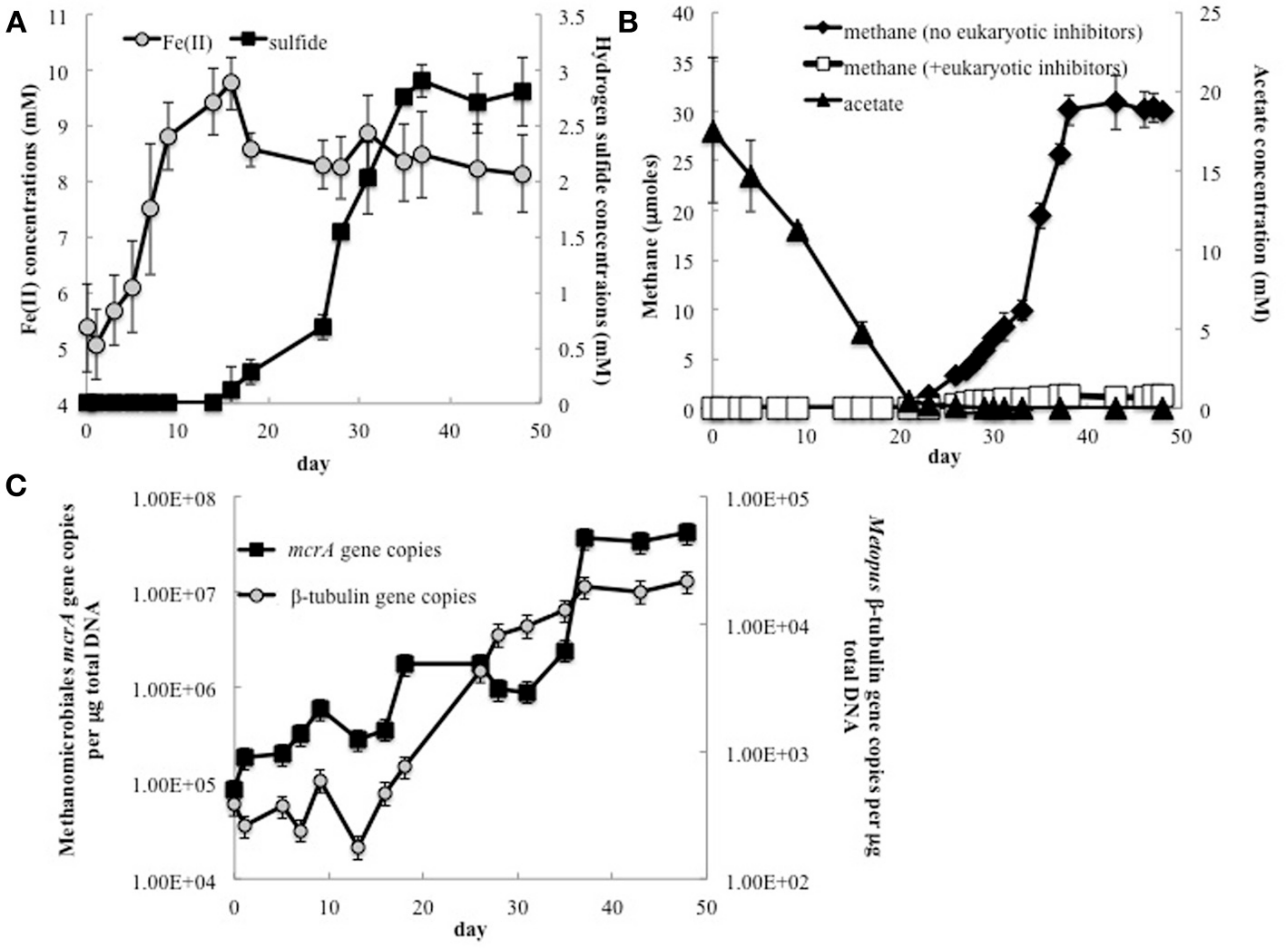
**(A)** Fe(II) and H_2_S concentrations in groundwater collected from Rifle sediment microcosms; **(B)** Methane and acetate concentrations in sediment incubations with and without addition of the eukaryotic inhibitors cycloheximide and colchicine; **(C)** The number of Methanomicrobiales *mcrA* and *Metopus* ß-tubulin gene copies detected in groundwater collected from sediment incubations.

In order to further evaluate the potential contribution of methanogenic endosymbionts to methane production, *in situ* uranium bioremediation was mimicked in anaerobic subsurface sediment incubations (Figure 5). Previous studies (Barlett et al., 2012a) have demonstrated that the addition of acetate to subsurface sediments incubated under anaerobic conditions results in microbiological and geochemical changes similar to those observed during *in situ* uranium bioremediation.

As expected (Anderson et al., 2003; Miletto et al., 2011; Barlett et al., 2012b), the pattern of Fe(II) and sulfide accumulation in the sediment incubations suggested that Fe(III) reduction was initially the predominant terminal electron accepting process, followed by sulfate reduction (Figure 5A). Methane production was not significant until acetate concentrations dropped below 275 μM and concentrations were ~2.5 times lower in the sediment incubations than they were in the field experiment (Figure 5B). This difference can be attributed to the fact that acetate was continuously pumped into the subsurface for the field experiments whereas acetate was only provided once at the beginning of the sediment incubation experiment.

Methanogenesis was significantly inhibited in sediments in which the eukaryotic inhibitors cyclohexamide and colchicine were added at the start of incubation (Figure 5B), suggesting that most of the methane production was related to protozoan activity, and presumably methanogens associated with these protozoa.

Analysis of gene copies for *Metopus* ß-tubulin, demonstrated that *Metopus* species began to increase in abundance as acetate was depleted (Figure 5C). After a brief lag this was followed by an increase in gene copies of clone 6 *mcrA*, the sequence that was correlated with *Metopus* species abundance in the field experiment. The factors responsible for the lag in the increase in the symbiotic methanogenic partner are not known. However, there was a direct correlation between Methanomicrobiales *mcrA* and *Metopus* ß-tubulin gene copies (*r* = 0.87, *p* = 0.0004), and between methane production and *Metopus* ß-tubulin (*r* = 0.97, *p* = 0.0003) and putative symbiont *mcrA* (*r* = 0.89, *p* = 0.02) gene copies.

## Discussion

These results demonstrate that stimulating *in situ* bioremediation of uranium-contaminated groundwater with the addition of high concentrations of acetate can have the unintended negative consequence of stimulating methanogenesis. Endosymbiotic methanogens harbored in protozoa appeared to be responsible for much of the methane produced after acetate additions stopped. These studies further emphasize the importance of protozoa in influencing microbial community dynamics and subsurface geochemistry during the bioremediation process.

Both the field experiment and laboratory incubation studies indicated that the enrichment of protozoa harboring endosymbiotic methanogens took place after acetate injections stopped and acetate concentrations had significantly declined in the aquifer. Previous studies showed that high concentrations of acetate stimulate the growth of Fe(III)-reducing and sulfate-reducing microorganisms, and specific protozoan populations that appear to specialize in predation of those actively growing microbes (Holmes et al., 2013). When acetate declined, the same rates of microbial growth could no longer be sustained and it is likely that moribund biomass then served as the primary energy source for a microbial community in which fermentative microorganisms became more important (N'Guessan et al., 2008). The lack of these ciliates or their symbionts at the site up-gradient of the acetate injection wells suggests that their prevalence following acetate injection is related to this increased availability of organic matter.

*Metopus* species are known to feed both on detritus as well as intact bacterial cells (Esteban et al., 1995; Dewdney, 2010). Although other protozoa also have this ability, protozoa such as *Metopus* species that possess symbiotic methanogens may have a competitive advantage as the growth rate of protozoa is increased when they harbor methanogens (Finlay and Fenchel, 1991; Biagini et al., 1998; Shinzato et al., 2007). The ciliate provides protection, energy and a carbon source for the methanogen, while the methanogen can excrete dissolved organics that can be used by the host (Nowack and Melkonian, 2010).

Fermentative microorganisms are also expected to be actively involved in degrading the biomass that accumulates following acetate injection (N'Guessan et al., 2008), producing H_2_ that can be consumed by free-living bacteria. This study showed that, without acetate additions to the subsurface, there was not sufficient electron donor supply to deplete sulfate from the groundwater (Figure 1B). Free-living methanogens cannot effectively compete with sulfate reducers for electron donors under these conditions (Lovley et al., 1982; Lovley and Klug, 1983). However, the endosymbionts have exclusive access to H_2_ produced within the hydrogenosome of the host protozoan. A comparable benefit to methanogens has been reported in marine sediments in which methanogen endosymbionts can account for 90% of the methane produced in the subsurface (Fenchel, 1993). In a similar manner, endosymbiotic methanogens produce a substantial amount of methane when rice paddy soils are first flooded and alternative electron acceptors such as Fe(III) and sulfate are available, but once Fe(III) and sulfate are depleted free-living methanogens account for most of the methane (Schwarz and Frenzel, 2005).

These results further demonstrate the important impact that protozoa can have on the ecology and biogeochemistry of *in situ* uranium bioremediation. It is likely that protozoa have similar influences in other bioremediation strategies that rely on stimulating microbial metabolism with organic electron donors.

### Conflict of interest statement

The Associate Editor declares that despite having collaborated with author Kenneth H. Williams, the review process was handled objectively. The authors declare that the research was conducted in the absence of any commercial or financial relationships that could be construed as a potential conflict of interest.
