# Supplementary material for: Methane production from protozoan endosymbionts following stimulation of microbial metabolism within subsurface sediments
Source: Front Microbiol. 2014 Aug 6;5:366. doi: 10.3389/fmicb.2014.00366 (PMC4123621; doi:10.3389/fmicb.2014.00366)
Supplement: Supplementary file 1 [file Presentation1.PDF]

Supplementary Figure S1. Orientation of the monitoring wells relative to the acetate injection gallery during the 2011 field experiment at the Rifle, CO study site.

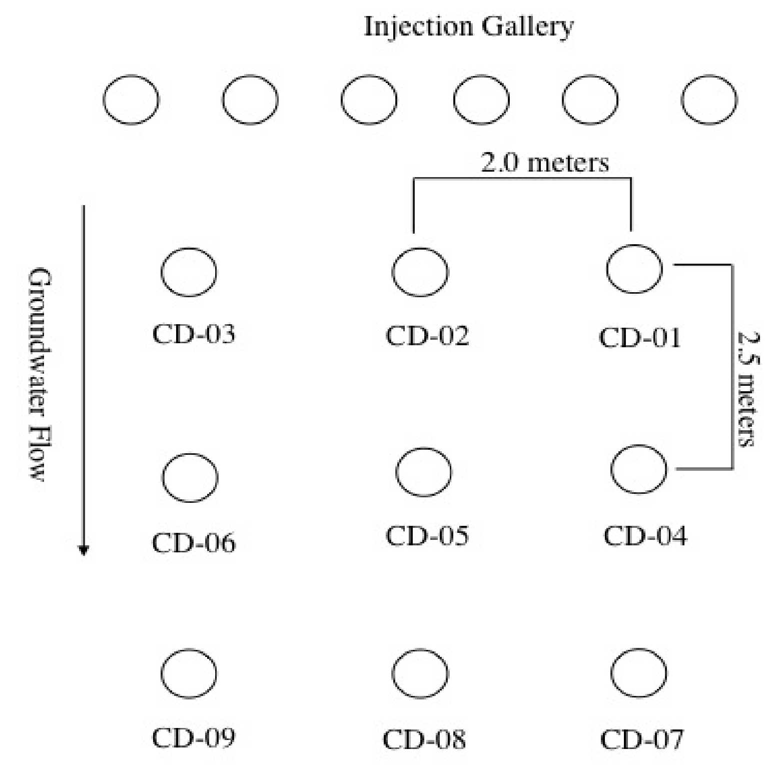

Supplementary Figure S2. The number of *Metopus* β-tubulin genes detected in the groundwater over the course of the 2011 field experiment.

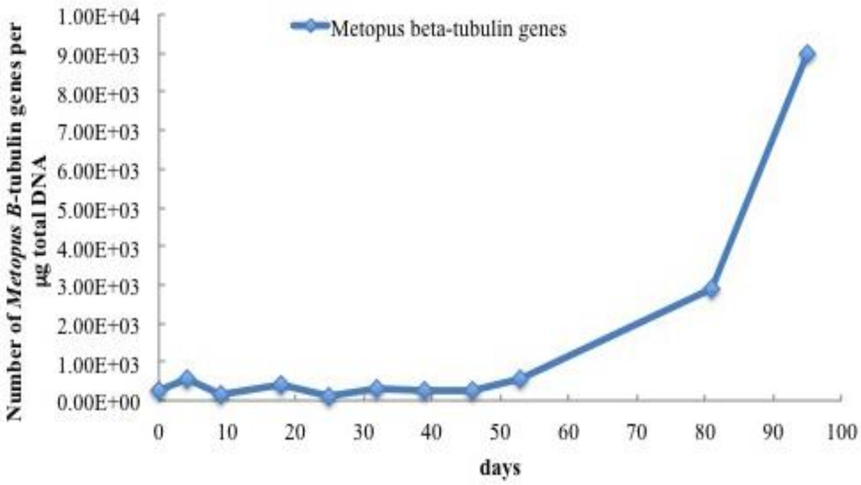

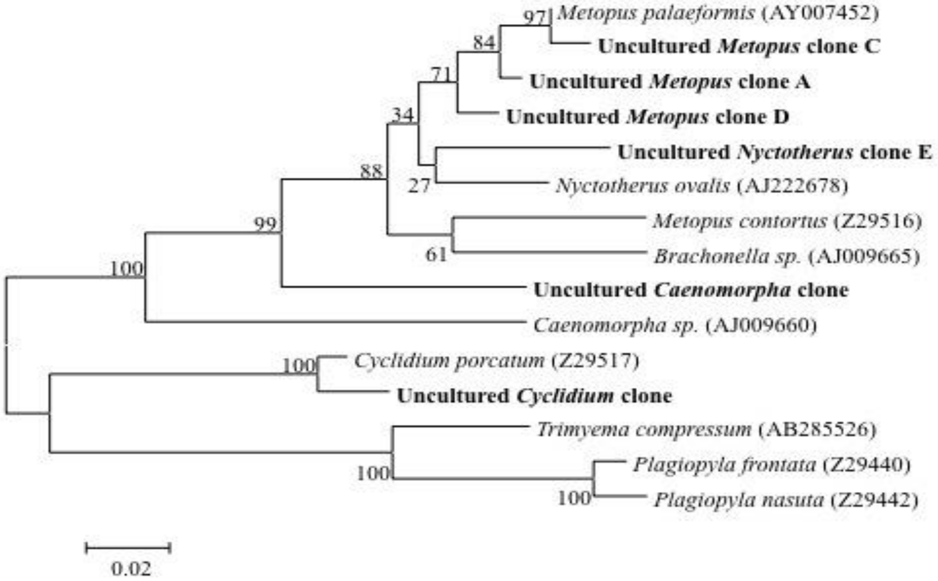

Supplementary Figure S3. Comparison of 18S rRNA gene sequences from protozoa detected in Rifle groundwater to sequences from protozoa known to harbor methanogenic endosymbionts. Phylogenetic tree constructed by the maximum likelihood method with 100 bootstrap replicates.

Supplementary Figure S4. Comparison of the dominant protozoan ß-tubulin mRNA sequence detected in cDNA libraries constructed from mRNA extracted from groundwater on day 95 of the field experiment to ß-tubulin sequences from other protozoan species. Nucleotide sequences were analyzed with the maximum likelihood method with 100 bootstrap replicates.

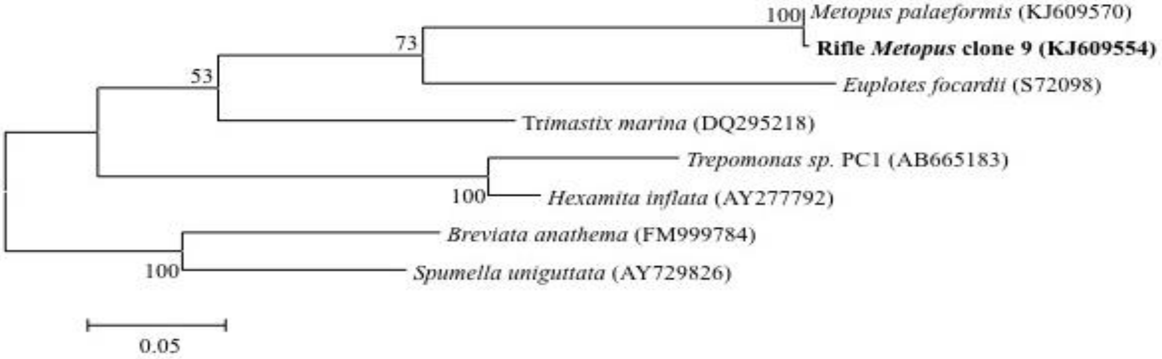

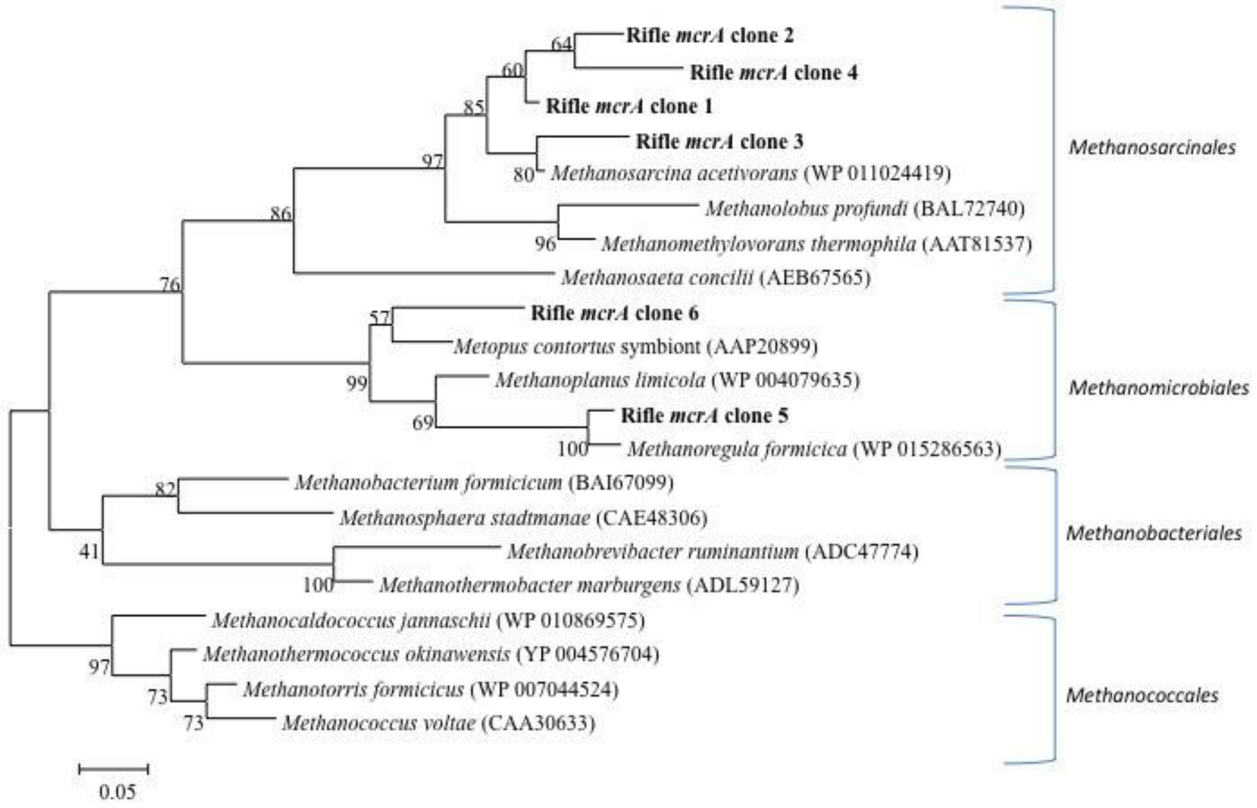

Supplementary Figure S5. Phylogenetic tree comparing *mcrA* mRNA transcripts detected in cDNA clone libraries assembled with RNA extracted from groundwater collected during the field experiment. Nucleotide sequences were analyzed with the maximum likelihood algorithm with 100 bootstrap replicates.
