# Supplementary material for: Methane production from protozoan endosymbionts following stimulation of microbial metabolism within subsurface sediments
Source: Front Microbiol. 2014 Aug 6;5:366. doi: 10.3389/fmicb.2014.00366 (PMC4123621; doi:10.3389/fmicb.2014.00366)
Supplement: Supplementary file 2 [file DataSheet1.PDF]

Supplementary Table 1. Primers used to amplify bacterial and archaeal 16S rRNA, eukaryotic 18S rRNA, archaeal *mcrA*, protozoan ß-tubulin, Methanomicrobiales *mcrA*, and *Metopus* ß-tubulin gene fragments from groundwater collected from field experiments and laboratory sediment incubations for clone library and quantitative PCR analyses.

| Gene target | Primer name | Forward primer | Reverse primer | reference |
|---|---|---|---|---|
| Bacterial 16S rRNA gene | 8f/519r | AGAGTTTGATCMTGGCTCAG | TTACCGCGGCTGCTGGC | (Eden et al 1991, Lane et al 1985) |
| Archaeal 16S rRNA gene | 344f/915r | ACGGGGYGCAGGCGCGA | GTGCTCCCCCGCCAATTCCT | (Casamayor et al 2002 changes in archaeal) |
| Eukaryotic 18S rRNA gene | 515F/1209R | GTGCCAAGCAGCCGCGGTAA | GGGCATCACAGACCTG | (Giovannoni et al 1988, Reysenbach et al 1992) |
| Protozoa beta tubulin gene | BT107F/BT261R | AACAACTGGGCIAAGGTYACTACAC | ATGAAGAAGTGGAGICGIGGGAA | (Baker et al 2004) |
| Archaeal *mcrA* gene | MLf/ME2 | GGTGGTGTMGGATTCACACARTAYGCWACAGC | TCATKGCRTAGTTDGGRTAGT | (Juottonen et al 2006, Luton et al 2002) |
| Rifle Methanomicrobiales *mcrA* gene | Rifle_mcrA_379f/489r | TCCAGTCGACTTTGCACTTG | GTGGTGTCGGATTCACACAG | This paper |
| Rifle *Metopus* ß-tubulin gene | Met-bt_60f/155r | GTCGTCCGAAAGGAAGCTGA | GAGGGTTCCCATTCCTGAGC | This paper |


Baker BJ, Lutz MA, Dawson SC, Bond PL, Banfield JF (2004). Metabolically active eukaryotic communities in extremely acidic mine drainage. *Applied and environmental microbiology* **70:** 6264-6271.

Casamayor EO, Massana R, Benlloch S, Ovreas L, Diez B, Goddard VJ *et al* (2002). Changes in archaeal, bacterial and eukaryal assemblages along a salinity gradient by comparison of genetic fingerprinting methods in a multipond solar saltern. *Environmental microbiology* **4:** 338-348.

Eden PA, Schmidt TM, Blakemore RP, Pace NR (1991). Phylogenetic analysis of *Aquaspirillum magnetotacticum* using polymerase chain reaction amplified 16S ribosomal RNA specific DNA. *International Journal of Systematic Bacteriology* **41:** 324-325.

Giovannoni SJ, Delong EF, Olsen GJ, Pace NR (1988). Phylogenetic group-specific oligodeoxynucleotide probes for identification of single microbial-cells. *Journal of Bacteriology* **170:** 720-726.

Juottonen H, Galand PE, Yrjala K (2006). Detection of methanogenic Archaea in peat: comparison of PCR primers targeting the mcrA gene. *Research in Microbiology* **157:** 914-921.

Lane DJ, Pace B, Olsen GJ, Stahl DA, Sogin ML, Pace NR (1985). Rapid determination of 16S ribosomal RNA sequences for phylogenetic analyses. *Proceedings of the National Academy of Sciences of the United States of America* **82:** 6955-6959.

Luton PE, Wayne JM, Sharp RJ, Riley PW (2002). The mcrA gene as an alternative to 16S rRNA in the phylogenetic analysis of methanogen populations in landfill. *Microbiology-Sgm* **148:** 3521-3530.

Reysenbach AL, Giver LJ, Wickham GS, Pace NR (1992). Differential amplification of ribosomal-RNA genes by polymerase chain reaction. *Applied and environmental microbiology* **58:** 3417-3418.
